# Using qualitative methods for a conceptual analysis of measures of health status and presenteeism prior to a mapping study

**DOI:** 10.1007/s11136-020-02570-x

**Published:** 2020-07-22

**Authors:** Cheryl Jones, Katherine Payne, Suzanne M. M. Verstappen

**Affiliations:** 1 Arthritis Research UK-MRC Centre for Musculoskeletal Health and Work, Southampton, UK; 2 Manchester Centre for Health Economics, The University of Manchester, Manchester, M13 9PL UK; 3 Arthritis Research UK Centre for Epidemiology, Division of Musculoskeletal & Dermatological Sciences, School of Biological Sciences, Faculty of Biology, Medicine and Health, The University of Manchester, Manchester Academic Health Science Centre, Manchester, UK; 4 NIHR Manchester Biomedical Research Centre, Central Manchester University Hospitals NHS Foundation Trust, Manchester Academic Health Science Centre, Manchester, UK

**Keywords:** Presenteeism, Autoimmune, Qualitative, Health-related quality of life, Conceptual validity, Mapping, Prediction, Health status

## Abstract

**Objectives:**

The inclusion of productivity in economic evaluations is a contentious issue. Methods are currently being developed to assess how it may feasibly be included for specific interventions, such as workplace interventions (WPIs), where productivity is a key outcome. Mapping (also called cross-walking or prediction modelling) may offer a solution. Prior to producing a mapping algorithm, it is recommended that the conceptual validity between ‘source’ and ‘target’ measures be understood first. This study aimed to understand the conceptual validity of two existing measures of health status (EQ-5D; SF-6D) and presenteeism to inform the potential for a subsequent mapping algorithm.

**Methods:**

A purposive sample of individuals who were currently working and had either rheumatoid arthritis (RA), ankylosing spondylitis (AS) or psoriatic arthritis (PsA). Individuals were recruited through support groups. Semi-structured telephone interviews were conducted until data saturation (no new emerging themes) was reached. Deductive and inductive framework analysis methods were used to identify key aspects of the conditions (themes) that impact on presenteeism (working at reduced levels of health).

**Results:**

Twenty-two (RA = 10; AS = 9; PsA = 3) employed individuals were interviewed. Deductive analysis identified evidence which confirmed the domains included in the EQ-5D and SF-6D capture those key aspects of RA, AS and PsA that increase presenteeism. Inductive analysis identified an additional theme; mental clarity, not captured by the EQ-5D or SF-6D, was also found to have a direct impact on presenteeism.

**Conclusions:**

The results of the study indicate conceptual validity of both health status measures to predict presenteeism. The next step is to develop a mapping algorithm for presenteeism.

**Electronic supplementary material:**

The online version of this article (10.1007/s11136-020-02570-x) contains supplementary material, which is available to authorised users.

## Introduction

Including productivity in economic evaluations of healthcare interventions is a contentious issue from both methodological and practical decision-making perspectives. Some jurisdictions, for example The Netherlands following guidance issued by The National Health Care Institute (Zorginstituut Nederland, ZIN), recommend assuming the societal perspective and encourage the impact on productivity to be included in economic evaluations [1]. In contrast, some jurisdictions actively discourage the inclusion of productivity in economic evaluations. In England, the National Institute for Health and Care Excellence (NICE) explicitly states the impact of productivity should be excluded [1, 2]. NICE’s discouragement is based on the premise that a healthcare budget is spent with the goal of increasing health and, therefore, only those costs and consequences that directly relate to the healthcare service should be included [3].

Normative arguments for the inclusion or exclusion of productivity are largely focussed on the distribution of consequences across different patient groups [4]. One view is how the inclusion of productivity may favour those interventions that predominately target the working population [5]. An alternative view suggests by neglecting to take account of productivity it may lead to unfavourable decisions for those interventions that help people who are struggling to stay at work or need help returning to work [6]. Methods developed to identify, measure and value the impact of presenteeism, suitable for economic evaluations, have focussed on quantifying the impact of presenteeism using costs. This approach means that wages must be used to value presenteeism which potentially introduces discrimination associated with factors such as gender, age, ethnicity, and educational attainment. Discrimination associated with wages forms the basis of arguments by Olsen and Richardson [5] against the inclusion of productivity in economic evaluations. An alternative conceptualisation of quantifying the impact of presenteeism is to view it as a non-monetary outcome, which would be of particular relevance for specific interventions that aim to improve both health status and subsequently ability to work (productivity).

A relevant example are workplace interventions (WPI), a set of activities, programmes or equipment that aim to help people with health conditions to remain or return to work by alleviating symptoms and/or improving functional ability [7]. WPIs, unlike many clinical interventions, may be funded by employers, or a healthcare service, with the goal of improving health and avoiding lost productivity [8].

Productivity is made up two distinct but related concepts: absenteeism and presenteeism. Absenteeism refers to the impact on productivity caused by being *absent* from work because of poor health [9]. Presenteeism identifies the impact on productivity while *at work* because of poor health [10]. Evidence suggests that the impact associated with presenteeism is far greater than that caused by absenteeism [11]. However, far less evidence exists that supports if, and how, presenteeism may legitimately be incorporated, measured and valued in economic evaluations of interventions, such as WPIs [12]. The apparent confusion over which methods ought to be used to capture the impact on presenteeism may have inadvertently discouraged researchers from collecting presenteeism-related data further limiting its availability to conduct further research [13]. Brouwer et al. [6] suggested it may be possible to predict presenteeism using health status data; two studies have begun to explore this by developing prediction models using regression methods; however, results are mixed and both conclude more research is required [13, 14].

Mapping techniques (cross-walking; prediction modelling), describe a set of methods that are used to generate a quantitative link between a ‘source’ and ‘target’ measure to predict unavailable outcomes using existing data [15]. Mapping has generally been used to develop algorithms that use data to link disease-specific non-preference-based measures to predict preference-based scores such as the EuroQol Five Domains (EQ-5D) [16, 17]. Mapping, while a second-best solution, allows researchers to estimate missing data keeping the burden on costs and time to a minimum. To date, mapping methods have not been used to develop an algorithm that links health status data with presenteeism; a potential method that may prove to be useful to predict levels of presenteeism associated with specific health states captured by generic measures of health status such as the EQ-5D. A number of measures of presenteeism are available [18, 9] an example includes the Work Productivity Activity Index (WPAI) [19], a short survey designed to ask patients about their ability to work recording both absenteeism and presenteeism. The idea would be to use health status data, captured by the EQ-5D or Short Form Six dimensions survey (SF-6D) [20], to predict levels of presenteeism measured by the WPAI. This paper reports on the findings of a study to derive a preference-based measure of health from the SF-36 for use in economic evaluation. The SF-36 was revised into a six-dimensional health state classification called the SF-6D. A sample of 249 states defined by the SF-6D has been valued by a representative sample of 611 members of the UK general population, using standard gamble. Models are estimated for predicting health state valuations for all 18,000 states defined by the SF-6D. The econometric modelling had to cope with the hierarchical nature of the data and its skewed distribution. The recommended models have produced significant coefficients for levels of the SF-6D, which are robust across model specification. However, there are concerns with some inconsistent estimates and over prediction of the value of the poorest health states. These problems must be weighed against the rich descriptive ability of the SF-6D, and the potential application of these models to existing and future SF-36 dataset.

Conceptual validity is defined as the extent to which the ‘content of two different instruments reflect one another when used for mapping’ [21]. Studies conducted to assess the conceptual validity between two measures prior to the development of a mapping algorithm or prediction model are limited meaning that some existing algorithms may produce biased estimates and lead to incorrect decisions regarding resource allocation [21]. Following Round and Hawton [21] recommendations, this study aimed to understand the conceptual validity supporting the use of measures of health status (EQ-5D; SF-6D) to predict the degree of presenteeism. The findings from this qualitative study could be used to inform the potential development of a mapping algorithm for presenteeism using generic multi-attribute measures of health.

There are a number of long-term health conditions that are known to affect the ability of people to work. Inflammatory autoimmune conditions are one example and used as the focus for this study. Rheumatoid arthritis (RA), ankylosing spondylitis (AS), and psoriatic arthritis (PsA) are three inflammatory autoimmune conditions that have been previously shown to affect ability to work [22–24]. RA affects women more than men [25], AS affects men more than women [26] and PSA affect men and women equally [27, 28]. There is substantial evidence to support that these conditions are responsible for a significant reduction in productivity in Europe, second only to mental health conditions [29]. These three conditions are the most common inflammatory autoimmune conditions in the United Kingdom (UK) affecting the body’s joints, tendons, muscles, and ligaments causing pain, stiffness, and fatigue of the joints [30]. If left untreated these conditions may cause permanent damage leaving the individual disabled [31]. Typically, the age of disease onset for all three conditions occurs before the age of 65 years old (the current retirement age in the UK meaning that individuals are affected during their working lifetime [25, 32, 33].

## Methods

### Study population and sample

The relevant study population was working individuals with RA, AS, or PsA. A working individual was defined as a person who is employed or self-employed, working full-time or part-time for pay. Individuals in voluntary roles were excluded from the study because the pressure to continue working at a high level are less pronounced. Individuals could not on be on sick leave at the time of the interview.

The study aimed to recruit a maximum of 30 working people (hereafter ‘employees’) with RA, AS or PsA taking into account data saturation. For qualitative analyses, sample size recommendations vary with some suggesting 20 to 30 [34] and others 30 to 50 [35]; however, in practice, the final sample size is determined by data saturation; the point at which no further concepts (themes are introduced. Recruitment and data collection was terminated once data saturation was met in this study. A purposive sample of employees were recruited using online advertisements on selected support group websites including the National Rheumatoid Arthritis Society, the National Ankylosing Spondylitis Society and the Psoriasis Association. The factors guiding the selection of individuals to invite for interview included trying to achieve an appropriate balance in the sample in terms of: years since diagnosis; gender; condition (RA, AS, PsA); type of work (manual or non-manual).

### Data collection

Semi-structured interviews were conducted by one researcher (CJ) over the telephone. The interview schedule was developed through discussions with two experts in presenteeism and inflammatory autoimmune conditions (Epidemiologist; Consultant in Occupational Rheumatology) and comprised three sections (see Online Appendix 1). The first section focussed on the patient’s rheumatic condition. The second section focussed on the types of tasks they complete in their current job. The third section asked respondents to discuss whether, and how, their rheumatic condition affects their ability to work. All interviews were conducted between June and September 2016.

### Data analysis

All interviews were recorded, transcribed and uploaded into the computer software package NVivo (version 11.0). Thematic content analysis, specifically the framework analysis method (FAM), is a frequently used approach to analyse data collected from semi-structured interview transcripts [36]. The FAM was used to identify common themes within the data that describe the key issues [37]. The data collected were analysed on an iterative basis alongside recruitment and continued until saturation was reached after which point no additional participants were recruited. Data were analysed in two stages using: (i) deductive and; (ii) inductive analysis methods.

The deductive analysis method was used to identify themes that were consistent with pre-existing theories or frameworks. This analysis was used to confirm or refute the conceptual validity of using existing measures of health status in terms of their ability to capture the impact of the selected inflammatory autoimmune conditions on presenteeism. The term health status is often interchanged with HRQoL in the literature and [38] offer a useful approach to delineate the appropriate use of the respective terms. In this study we used the term health status, rather than HRQoL, as the focus is on available surveys used to measure specific states of health rather than the resulting value (or utility) of HRQoL.

Two existing health status measures were selected as the existing framework to guide the deductive analysis. The EQ-5D was selected because it is currently recommended by NICE as the preferred measure of health status for use in economic evaluations of healthcare technologies and diagnostics [39, 40]. The SF-6D, an alternative generic measure of health status, was selected because it can be derived from the SF-36 [41] which is a commonly used survey in populations with inflammatory arthritis [42].

Table 1 lists the domains included in the EQ-5D and SF-6D to describe and define an individual’s health status. The domains included in the EQ-5D and SF-6D provided the pre-defined themes which were taken to the data to understand whether there was evidence that the domains included in the two health status measures were important factors that cause presenteeism due to RA, AS or PsA. The participants were not asked to complete the EQ-5D or SF-6D as part of the interview to avoid introducing researcher bias which could arise because their answers would be influenced as a result of having just completed a health status questionnaire.

**Table 1:** Domains and definitions included in two measures of health status (the SF-6D and the five-level version of EQ-5D)

Health status measure	Dimensions/concept theme	Definition
EQ-5 D^a^	Mobility	How well can the individual walk around
	Self-care	Can the individual wash or dress themselves
	Usual activities	How well can the individual work, study, do housework, spend time with family or do leisure activities
	Pain/discomfort	How much pain or discomfort is a person experiencing
	Anxiety/depression	How anxious or depressed is the individual feeling
SF-6D^b^	Physical functioning	Does the individual’s health limit their ability to do vigorous activities, moderate activities, or bathing or dressing?^1^
	Role limitation	Does the individual have problems with work or carrying out other daily activities because of physical or emotional health problems?
	Social functioning	Does the individual’s health problem limit their social activities?^2^
	Pain	How much pain interferes with the individual’s ability to do both work inside and outside of the home?
	Mental health	How often does the individual feel tense or downhearted?
	Vitality	How often does the individual feel they have lots of energy?

*Source:* ^a^Gudex et al. [43] and ^b^Brazier et al. [20]

^1^Vigorous activities is defined as running, lifting heavy objects, participating in strenuous sports. Moderate activities is defined as moving a table, pushing the vacuum cleaner, bowling or playing golf

^2^Social activities include visiting friends and family

The definitions of each domain for each health status measure (Table 1) provided by the original studies were used to determine whether the domains conceptually overlapped. For example, the SF-6D includes ‘physical functioning’, defined as ‘vigorous’ or ‘moderate activities’, or ‘bathing and dressing’, which conceptually overlap with ‘mobility’ and ‘self-care’ domains included in the EQ-5D. In preparation for the deductive analysis, where distinct and independent concepts are needed prior to analysis, both measures were analysed to identify overlapping concepts and to generate independent themes.

In situations where the deductive approach is used qualitative researchers also recommend performing an inductive analysis to ensure no further important themes are missed [36]. The inductive analysis method was used to analyse the data to identify new themes that may impact on presenteeism but are not captured by existing frameworks represented by the two health status measures (EQ-5D and SF-6D). Further information about the analysis approach used is presented in Online Appendix 2.

## Results

A total of 22 employees were interviewed (see Table 2). The majority of the study sample were females (82%) working in non-manual roles with RA or AS. The number of years since diagnosis ranged from 1.5 to 32 years; however, of the 18 employees who reported the year of their diagnosis, 61% reported they had their condition for less than 10 years. Some employees (*n* = 4) were not able to recall the time of their diagnosis.

**Table 2:** Characteristics of the study sample of individuals working with RA, AS or PsA

Characteristics	Number of interviewees (*n* = 22)
Gender	
Male	4 (18%)
Female	18 (82%)
Condition	
Rheumatoid arthritis	10 (45%)
Ankylosing spondylitis	9 (41%)
Psoriatic arthritis	3 (14%)
Years with disease since diagnosis	
Less than 5 years	8 (36%)
Between 5 and 10 years	3 (14%)
Between 11 and 20 years	5 (23%)
More than 20 years	2 (9%)
Missing data	4 (18%)
Current medication	
csDMARDs	5 (22%)
Biologics	12 (54%)
Other (NSAIDs)	3 (14%)
None	1 (5%)
Missing data	1 (5%)
Reported effective medication	
Yes	17 (77%)
No	1 (5%)
Not sure	1 (5%)
Missing data	2 (9%)
Job type^a^	
Manual	5 (23%)
Non-manual	17 (77%)

*csDMARDS* conventional synthetic disease-modifying anti-rheumatic drugs

^a^Job type was defined by the tasks reported by the individual during the interview. Manual roles were assigned to individuals who described job tasks that required a substantial physical demand

### Deductive analysis results

**Fig. 1:**
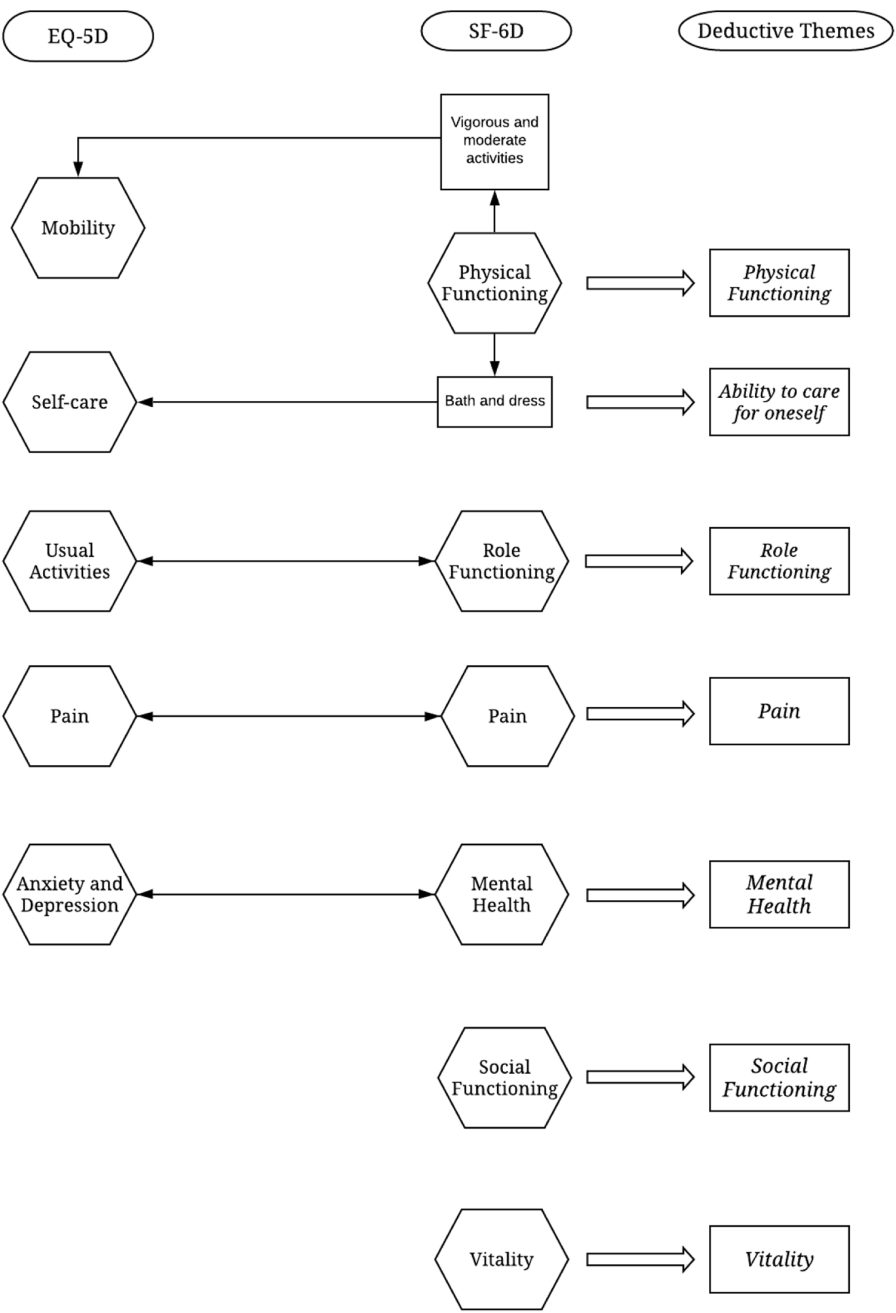
Independent themes for deductive analysis

The first step in the deductive analysis was to summarise instances in which the states of health described in the surveys conceptually overlap (see Fig. 1) between the two measures of health status and collate the number of distinct themes. The conceptual overlap summarises the instances in which the domains included in the EQ-5D also feature in the SF-6D, of which there were four. Two further concepts captured in the SF-6D, social interaction and vitality (fatigue), were not included in the EQ-5D. A total of seven independent (deductive) themes were identified for the deductive analysis and are reported in Fig. 1.

The deductive analysis identified evidence, emerging from the interviews, consistent with the independent themes included in the EQ-5D and SF-6D. These seven themes are now described in more detail in terms of how each aspect of health status impacted on the ability of individuals with inflammatory autoimmune conditions to work.

#### Physical functioning

Physical functioning is captured by the domain with that label in the SF-6D and also by the domain ‘mobility’ in the EQ-5D. Participants working in non-manual jobs frequently stated that long meetings were difficult because they struggle to sit for that length of time without becoming very uncomfortable. Participants also agreed that stiffness of the joints was problematic and usually worse in the morning making it difficult for individuals to prepare for work that starts early.

my main concern at the moment is that I may have to pull out of this [driving] course because I don’t feel safe to drive (P101, AS, female)

physically going round their [farmers] premises [farms]…there are one or two that I go to where we have a meeting in the house and discuss financial stuff, which is fine, that’s manageable, but I can’t go traipsing round fields (P104, RA, female)

#### Ability to care for oneself

Ability to care for oneself is captured by the domain labelled ‘self-care’ in the EQ-5D and is synonymous with the level ‘bath and dress’ within the physical functioning domain in the SF-6D.

Some participants reported that they found it difficult to get washed and dressed quickly in the morning making it difficult to get ready for work on time without having to get out of bed very early; one participant explained that it was often easier for her to work from home to avoid the stress of getting ready early in the morning and being able to get extra rest in bed. Those participants who could not work from home explained they have to get up extra early to have enough time to prepare themselves for work forcing them to reduce hours of sleep and ultimately contributing towards increased fatigue.

fact is you’ve got to get yourself ready to go to work, that would be awful. I mean, as it is, I can just haul myself out of bed and sit down here in my pyjamas and carry on working and answer my phone (P104, RA, female)

I start work officially at 9 o’clock, but I have to get up at 7 in the morning to take my first lot of painkillers for them to kick in before I go to the bathroom to start getting washed and dressed, which is about 8 o’clock (P112, AS, female)

#### Role functioning

Role functioning is captured by the domain with that label in the SF-6D and in the domain labelled ‘usual activities’ in the EQ-5D. Participants who work in non-manual jobs stated that they struggle to carry out usual activities, such as writing and typing, which negatively impacts the quantity and quality of work they produce. Participants also explained that, because of their condition, they feel obliged to work extra hours to in order to catch up. Participants working in manual jobs stated that they struggle with the physical aspects of their jobs, such as heavy lifting, bending and moving quickly; limiting what they are able to achieve during the working day.

it’s very bad and everything is very stiff, urm I can have trouble walking or I can have trouble using my hands and a lot of the work involves typing (P108, AS, female)

its [AS] fused my lower back now, so I do struggle to pick up objects off the floor… I fit windows and doors, skirting boards, things, for kitchens, urm..ur…flooring, I do, which [laughs] as you can imagine, is a struggle (P107, AS, male)

#### Pain

Pain is a distinct domain in the EQ-5D and SF-6D. The majority of the participants stated that they constantly felt some level of pain which had a direct impact on their ability to work. Some said their pain was initially the most difficult symptom to cope with; however, others said that they have adapted and, to some extent, could ignore it but admit reliance on analgesics.

I was in agonising pain….it did affect my [me] day-to-day, I’d struggle, as I said, to write, to type, I put that I had to have someone typing for me (P110, PsA, female)

[I] was constantly in pain, I could hardly sleep, urm… yeah, it’s completely changed, I’ve had to change jobs twice and yeah…completely changed my life (P111, AS, female)

#### Mental health

Mental health is captured by the domain with that label in the SF-6D and in the domain labelled ‘anxiety and depression’ in the EQ-5D. The majority of the participants explained that their condition negatively affected their emotions and can made it difficult to achieve the same amount of work compared to days where they generally feel happier.

on a good day, I can give it my all and energy and feel happy and almost normal…that’s the difference (P106, AS, male)

Mood is better, happier, joining in with other people, general chit-chat, and then, you know, all of a sudden it’s the end of the day and time to go home, you know, keeping myself busy, doing a lot of things (P123, AS, female)

#### Social functioning

Social functioning is captured by the SF-6D. Participants felt that the condition prevents them from being able to interact positively with work-colleagues and consequently are more likely to isolate themselves. The inability to be as sociable at work meant, for some participants particularly in management positions, it was more difficult to motivate staff or engage enthusiastically in meetings. Many participants also reported that they are more likely to avoid contact with colleagues who have come to work with contagious illnesses (common cold) because of their reduced immune system due to medication used for RA, AS or PsA.

on a good day, I’m all smiley and happy, I can walk round, engage with my colleagues and get them to do what I want to do, but not anymore really… (P106, AS, male)

because you have a hardly any immune system, you have to swerve people when they’re ill and they think you are being precious…. socially, it can cause a bit of agro, because I will say to people ‘will you go away, because if I catch that I’ll be really poorly and it’ll take me ages to shake it off’ (P103, AS, female)

#### Vitality

Vitality is measured by the SF-6D. Lack of vitality frequently described as the symptom that was most difficult to work with, cope with, adapt to, and manage. In terms of being able to complete tasks for their job, participants reported that they struggled with the effects of fatigue and said they need more energy to work effectively irrespective of job type. As a result, some participants stated they had to reduce the number of hours/days they work per week or changed jobs entirely.

I had to give up being a solicitor in private practice because I was too knackered, I was just exhausted all the time and had brain fog and couldn’t function, and I just had to step away from that entirely (P108, AS, female)

to be fully effective in my job I would need to be able to write a lot more than I can and I would need to have more energy to do extra things (P109, RA, female)

### Inductive analysis results

The inductive analysis identified one additional theme, ‘mental clarity’ not captured by the EQ-5D or SF-6D and affecting levels of presenteeism. Mental clarity (more often referred to as ‘brain fog’ by the participants) describes an individual’s inability to concentrate or become forgetful but *not* because of emotions linked to mental health. Nearly half of the interviewees used the term ‘brain fog’ to describe how they were limited in terms of their ability to concentrate and think quickly slowing the speed at which they can work do their job, increasing the probability of making mistakes, and forgetting to complete tasks. Participants working in both manual and non-manual roles reported to be affected by ‘brain fog’.

yeah there is the concentration part, I keep mentioning this brain fog thing, where […] you’re concentrating but it’s difficult to think of too many things at once….like today, I’ve, sort of, forgotten a few things and it’s been pulled up (P113, AS, male)

my nightmare days are when I’ve got a report to write and I can’t concentrate, I’ll have written a paragraph and an hour later I’ll still be looking at it or reading through something that I’ve already read 3 times because the concentration (P114, RA, female)

## Discussion

This study provided evidence using qualitative methods to support the conceptual validity of using two measures of health status (EQ-5D and SF-6D) to capture the impact on presenteeism caused by chronic health conditions, such as RA, AS and PsA. Overall, the qualitative evidence showed the domains within both multi-attribute measures of health status conceptually overlapped with the elements of poor health that resulted in presenteeism. This support for conceptual validity of the two existing measures of health status suggested they could be used as potential source measures in a subsequent study to produce a mapping algorithm to predict presenteeism. Directly comparing the conceptual validity of the two measures indicates that the SF-6D may be a more suitable measure to predict presenteeism because it includes two relevant constructs: ‘social interaction’ and ‘vitality’ (fatigue). A qualitative study of a working sample of employees with RA, AS or PsA found fatigue potentially has the greatest impact on presenteeism [44, 45]. However, neither of the two measures of health status captured the impact of low ‘mental clarity’ on ability to work. Mental clarity, in the exemplar conditions, was a particularly important symptom preventing individuals from being able to think quickly and/or increasing the number of mistakes they made in their work.

This is the first qualitative study that has produced empirical evidence to understand the conceptual validity of ‘source’ and ‘target’ measures, in this case EQ-5D and SF-6D with presenteeism, prior to the development of a mapping algorithm. The study was conducted based on those recommendations published by Round and Hawton [21]. The results of this study provide evidence that supports the conceptual validity of using measures of health status, as measured by the EQ-5D and SF-6D, to predict levels of presenteeism, measured using for example the WPAI. These findings provide motivation to develop a subsequent mapping algorithm, which will be the next steps.

Two published prediction models for presenteeism that use data from a measure of health status, the EQ-5D-3L, provide some evidence that a quantitative relationship linking health status with the impact on presenteeism may exist; however, the evidence was insufficient to recommend the estimated prediction model [13, 14]. Given these existing studies, the evidence from this qualitative study exploring how measuring health status could capture the impact on presenteeism provides additional insights to understand the identified ‘weak’ quantitative relationship linking health status and presenteeism. Using qualitative methods allows some explanation and interpretation for quantitative results derived from a subsequent mapping algorithm.

### Limitations

The results from this study only support a positive (descriptive) stance suggesting that the SF-6D, is potentially, more likely to be a suitable measure to capture presenteeism compared with the EQ-5D. A subsequent quantitative study that produces a mapping algorithm to measure the association between measures of health status and presenteeism could be used to provide further evidence to answer a normative question. The next step is to assess the predictive ability of the EQ-5D and SF-6D to levels of presenteeism using quantitative (regression) methods.

The inductive analysis identified ‘mental clarity’ as an additional symptom of RA, AS and PsA that impacts presenteeism. This finding can be viewed in two ways. It could be argued that this limits the generalisability of the findings from this study to other conditions. An alternative interpretation is that it is known that other chronic conditions, such as chronic fatigue syndrome, fibromyalgia, migraines [46, 47], also report difficulties with mental clarity, which would suggests the findings maybe be generalisable to other conditions. A further study in other conditions is needed to provide evidence of the relevance of mental clarity on the impact of working with other health conditions.

An opportunity was missed to collect EQ-5D, SF-6D and WPAI scores after the qualitative interview was conducted. Collecting such data may have provided some interesting insights about how individuals describe how their health affects their ability to work compared with how they might report their health status on that day or level of presenteeism over the past 7 days (WPAI). However, on balance we felt the additional cognitive burden and potential for introducing research bias by asking respondents to complete these outcome measures outweighed the potential value of collecting a small sample of responses.

The participants interviewed for this study worked in manual and non-manual jobs. A relatively small proportion of the sample interviewed worked in manual jobs, which may limit generalisability of the results to those working in these occupations. However, the results, arguably, reflect the UK, and other developed countries, where a large proportion of working people predominantly work in non-manual jobs [48]. The study was only able to recruit and interview a relatively low proportion of men, which may limit generalisability of the impact inflammatory autoimmune conditions have on men and their ability to work. However, the responses from both genders within this study were similar and there is little reason to believe that the results would have been substantially different had more men been included.

The focus of this study was to understand which of the domains within existing outcome measures of health status are most relevant to capture the impact of inflammatory arthritis on ability to work. The degree of the impact, which is likely to be associated with the severity of disease experienced by the working individual, could potentially be captured by the levels attached to each domain within the outcome measures. We did not explore this issue which could be the topic of a subsequent study that aimed to understand how disease severity influences presenteeism.

## Conclusion

This study supports the use of qualitative methods to understand the conceptual validity of source measures before developing a mapping algorithm or prediction model. The conceptual validity of using two existing multi-attribute measures of health status (EQ-5D and SF-6D) was sufficient to suggest their use as source measures to predict presenteeism (measured by the WPAI) in working people with RA, AS or PsA. Both health status measures will be taken forward as key predictors for the development of a mapping algorithm that links health status with presenteeism.

## Electronic supplementary material

Below is the link to the electronic supplementary material.

Supplementary file1 (DOCX 22 kb)
