# Supplementary material for: Using qualitative methods for a conceptual analysis of measures of health status and presenteeism prior to a mapping study
Source: Qual Life Res. 2020 Jul 22;29(11):3167–77. doi: 10.1007/s11136-020-02570-x (PMC7591427; doi:10.1007/s11136-020-02570-x)
Supplement: Supplementary file 1 — Supplementary file1 (DOCX 22 kb) [file 11136_2020_2570_MOESM1_ESM.docx]

**Supplementary Appendices for: Using qualitative methods for a conceptual analysis of measures of health status and presenteeism prior to a mapping study**

Cheryl Jones, Ph.D,^1,2*^, Katherine Payne, Ph.D^1^ _,_ Suzanne M.M. Verstappen, Ph.D^3,4^

^1^ Manchester Centre for Health Economics, The University of Manchester, Manchester, United Kingdom

^2^ Arthritis Research UK-MRC Centre for Musculoskeletal Health and Work

^3^ Arthritis Research UK Centre for Epidemiology, Division of Musculoskeletal & Dermatological Sciences, School of Biological Sciences, Faculty of Biology, Medicine and Health, The University of Manchester, Manchester Academic Health Science Centre, Manchester, United Kingdom

^4^ NIHR Manchester Biomedical Research Centre, Central Manchester University Hospitals NHS Foundation Trust, Manchester Academic Health Science Centre, UK

**Supplementary Appendix 1: Semi- structured Interview Schedule**

**Supplementary Appendix 2: Deductive and inductive qualitative analysis methods**

**Supplementary Appendix 1: Semi- structured Interview Schedule**

**Section A: Health Details**

1. Please tell me what rheumatic/arthritic condition you have been diagnosed with?
   1. When were you diagnosed?
   2. How long did it take to be diagnosed?
   3. Are you taking any medication for your arthritis at the moment?
   4. What are you taking?
   5. Do you think it is effective?
2. In your opinion, how severely does your condition affect you day-to-day?
3. In terms of your condition, do you experience ‘good’ days and ‘bad’ days?

- *If yes;*
  1. How does a ‘bad’ day differ in terms of their effect on your day-to-day activities compared to a ‘good’ day?
  2. How often do you experience ‘bad’ days?
  3. How would you describe the progression of your disease? *(increasing over time, stable, in remission)*
- *If No;*
  1. How would you describe the progression of your disease? *(increasing over time, stable, in remission)*

**Section B: Job Details**

The following questions are about your current job

1. What is your job title?
2. How would you describe the type of industry that you work in? (for example construction, retail)
3. During a typical working day, what tasks do you perform?
4. Do you work as part of a team?

- *No;*

1. You work independently throughout the working week?

- *Yes;*

1. How many people work in your team?
2. Do you manage anyone in this team?
3. How many people are you managed by?
4. On those days you are absent from work, can your colleagues fill in for you and complete some of your work?

- *No;*

1. What happens to your work?

- *Yes;*

1. Who is able to do your work whilst you are away?
2. How much of your work can be done by your colleagues?

**Section C: Health and Work Performance**

1. Bearing in mind your condition, do you feel you are able to achieve what you want to achieve at your job? *(for example, to gain a promotion or to do your job to the best of your ability)*

- *Yes;*
  1. Are there any aspects of your job or workplace that help you to achieve your goals?
- *No;*
  1. What do you feel limits your ability to achieve your goals at work?

1. You mentioned earlier that you experience ‘good’ days and ‘bad’ days, in terms of your condition. How does your condition affect your ability to work on a ‘bad’ day compared to a ‘good’ day?

- Does your condition affect you at work when you are experiencing a ‘good’ day? If so, how?

1. Does your condition reduce the amount of work you can complete in a given amount of time?
   - *No;*
   - *Yes;*
2. What happens to the work that is not completed? For example;
   1. Work overtime
   2. Colleagues take work to complete
   3. Nothing – output is reduced
3. Does your condition reduce the quality (as opposed to quantity) of work you are able to produce?
   - *No;*
   - *Yes;*
     1. In what ways do you think your quality of work is reduced?
4. Has your condition ever compromised the safety of yourself or others at your workplace?
   - *No;*
   - *Yes;*
     1. Can you describe what happened?
5. In the past, have you had to change your job or alter your job, for example the hours you work, the types of tasks you complete, because of your condition?

- *No;*
- *Yes;*
  1. Can you please describe what happened?
  2. The changes that were made to your job because of your condition, how did they help you work more effectively?

1. Imagine you are able to make changes or further changes to your current workplace or job that would help you to manage your condition and allow you to work more effectively, what would you change? *(for example, starting work at 10am, going to the gym at lunch, raising my desk and standing to do work)*
2. Have you told any person at work (colleagues or managers) about your rheumatic condition?

- *Yes;*
  1. Who did you tell? *(no names, please describe as colleague or manager)*
  2. What made you decide to tell this person? *(trust, supportive workplace)*
  3. What was their reaction?
- *No;*

1. What has stopped you from telling someone at work about your condition?
   - 1. What would help you feel confident to tell your colleagues or manager about your condition?

**Work Performance and Other Issues**

1. Other than your rheumatic condition, are there any other issues you believe affect your ability to work effectively?

- *No; go to next section*
- *Yes;*

1. Please describe what they are
